# Supplementary material for: Splice-Junction-Based Mapping of Alternative Isoforms in the Human Proteome
Source: Cell Rep. Author manuscript; Available in PMC 2020 Jan 15. (PMC6961840; doi:10.1016/j.celrep.2019.11.026)
Supplement: 2 [file NIHMS1546469-supplement-2.zip › DF1/PXD006675/PulmonaryArtery/PulmonaryArtery_25_TAF1C_QVVTCTVQGENFQEVVLTPGDNP.pdf]

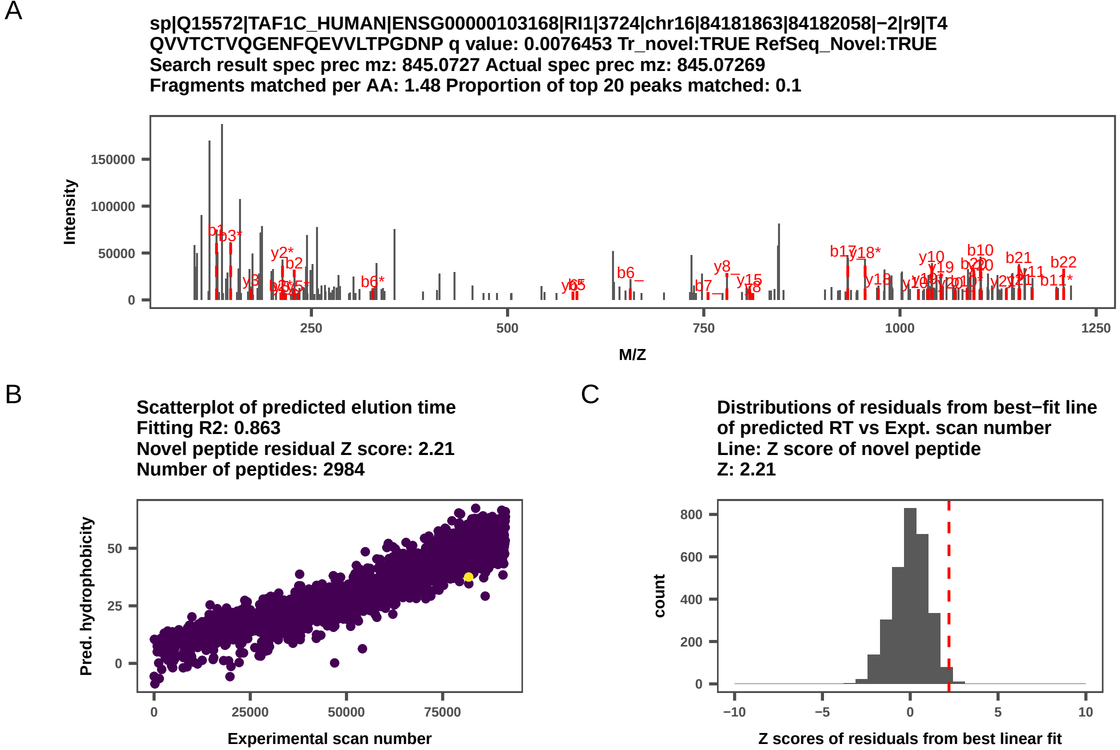
A
sp|Q15572|TAF1C_HUMAN|ENSG00000103168|RI1|3724|chr16|84181863|84182058|−2|r9|T4
QVVTCTVQGENFQEVVLTPGDNP q value: 0.0076453 Tr_novel:TRUE RefSeq_Novel:TRUE
Search result spec prec mz: 845.0727 Actual spec prec mz: 845.07269
Fragments matched per AA: 1.48 Proportion of top 20 peaks matched: 0.1
Intensity
150000
100000
50000
0
250
500
750
1000
1250
M/Z
b3*
y2*
b2
y3
b6*
b6
y8
y15
y18*
y10
b10
b21
b22
y18
y11
b11*
B
Scatterplot of predicted elution time
Fitting R2: 0.863
Novel peptide residual Z score: 2.21
Number of peptides: 2984
Pred. hydrophobicity
50
25
0
0
25000
50000
75000
Experimental scan number
C
Distributions of residuals from best−fit line
of predicted RT vs Expt. scan number
Line: Z score of novel peptide
Z: 2.21
count
800
600
400
200
0
−10
−5
0
5
10
Z scores of residuals from best linear fit
